# Spectrum Sensing Method Based on STFT-RADN in Cognitive Radio Networks

**DOI:** 10.3390/s24175792

**Published:** 2024-09-06

**Authors:** Anyi Wang, Tao Zhu, Qifeng Meng

**Affiliations:** School of Communication and Information Engineering, Xi’an University of Science and Technology, Xi’an 710054, China; 23207223061@stu.xust.edu.cn (T.Z.); p493237439@163.com (Q.M.)

**Keywords:** spectrum sensing, short-time Fourier transform, attention mechanism, dense connections, shortcut connections, cognitive radio networks

## Abstract

To address the common issues in traditional convolutional neural network (CNN)-based spectrum sensing algorithms in cognitive radio networks (CRNs), including inadequate signal feature representation, inefficient utilization of feature map information, and limited feature extraction capabilities due to shallow network structures, this paper proposes a spectrum sensing algorithm based on a short-time Fourier transform (STFT) and residual attention dense network (RADN). Specifically, the RADN model improves the basic residual block and introduces the convolutional block attention module (CBAM), combining residual connections and dense connections to form a powerful deep feature extraction structure known as residual in dense (RID). This significantly enhances the network’s feature extraction capabilities. By performing STFT on the received signals and normalizing them, the signals are converted into time–frequency spectrograms as network inputs, better capturing signal features. The RADN is trained to extract abstract features from the time–frequency images, and the trained RADN serves as the final classifier for spectrum sensing. Experimental results demonstrate that the STFT-RADN spectrum sensing method significantly improves performance under low signal-to-noise ratio (SNR) conditions compared to traditional deep-learning-based methods. This method not only adapts to various modulation schemes but also exhibits high detection probability and strong robustness.

## 1. Introduction

With the widespread adoption of 5G communication and the development of 6G technology, the era of the Internet of Everything and intelligent connectivity is approaching [1]. However, the rapid increase in wireless devices and the static management of the radio spectrum have led to a shortage within the available spectrum [2]. CRN is an effective solution to this spectrum resource scarcity [3]. The concept of CRN was introduced by Mitola in 1999. CRN is an intelligent wireless communication system that allows secondary users (SUs) to utilize the licensed spectrum without interfering with primary users (PUs). Spectrum sensing is a key technology in CRN, enabling SUs to determine in real time whether a frequency band is occupied by a PU, thereby efficiently utilizing the available spectrum [4].

Traditional spectrum sensing methods encompass energy detection [5], cyclostationary feature detection [6], matched filter detection [7], and eigenvalue-based detection [8]. These methods share the common characteristic of computing detection statistics and comparing them with a predetermined threshold to make a final decision. The accuracy of the detection statistics and thresholds directly affects the detection results. Traditional methods often perform poorly under low SNR conditions.

To overcome the challenge of determining detection thresholds in traditional spectrum sensing, researchers have increasingly proposed deep-learning-based methods. Deep learning excels at classifying large datasets, and spectrum sensing can be viewed as a binary classification problem to determine the presence or absence of a PU signal in complex wireless data. Compared to traditional machine learning methods, deep-learning-based spectrum sensing provides higher accuracy and robustness in complex wireless environments and high-interference conditions, enhancing its effectiveness [9]. CNNs are effective for extracting target features and outperform other machine learning algorithms in extracting two-dimensional image features [10]. Pan et al. [11] proposed a spectrum sensing method based on cyclostationary features and a CNN, which significantly improved detection probability compared to traditional methods. However, this method still has limitations, such as not being applicable to all modulation signals, insufficient feature extraction capabilities of the CNN, and gradient vanishing issues with deeper networks. Liu [12] and Han [13] used sample covariance matrices and cyclostationary and energy features as detection statistics and network inputs. These methods are applicable to various modulation signals and enhance detection probability by preprocessing time-domain signals, but traditional CNNs struggle to effectively extract features under low SNR conditions. Zheng et al. [14] proposed a ResNet-based spectrum sensing method that enhanced detection performance over traditional CNN. Both Cai [15] and Gai [16] employed STFT for signal preprocessing, leading to significant performance improvements. However, their approaches are still hindered by the limitations of a simple CNN architecture, which is inadequate for effective feature extraction. Wang et al. [17] proposed the RDN-CBAM method, significantly improving spectrum sensing performance under low SNR conditions by employing dense connections and integrating the CBAM attention mechanism. However, it still falls short in adequately representing signal features.

To overcome these limitations, we propose a more effective spectrum sensing method. By applying the STFT for the time–frequency analysis of received signals, we transform one-dimensional signals into two-dimensional time–frequency spectrograms, capturing the signal’s characteristics more comprehensively. Thus, we convert the spectrum sensing problem into an image binary classification problem. To enhance the feature extraction capability of traditional CNN and address gradient vanishing issues in deeper networks, we improve the basic residual block and introduce CBAM, combining dense and residual connections to propose RADN. This significantly enhances the network’s feature extraction capability. Combined with STFT, this forms the STFT-RADN spectrum sensing method.

## 2. System Model

In CRN, spectrum sensing is essentially a signal detection problem with two possible outcomes: the presence or absence of the primary user. This is similar to binary detection; thus, spectrum sensing can be transformed into a binary classification task. The binary sensing model is described as follows:

(1) $$\begin{array}{l} H_{0}:y(n)=g(n)\\ H_{1}:y(n)=s(n)+g(n) \end{array}$$

where $H_{0}$ indicates the absence of the PU, $H_{1}$ indicates the presence of the PU, n is the time series of the discrete signal, y(n) is the received signal, s(n) is the signal transmitted by the PU through a Rayleigh fading channel, and g(n) is the Gaussian noise with a mean of 0 and variance of $\sigma^{2}$.

The performance of spectrum sensing is typically evaluated using two metrics: detection probability ($P_{d}$) and false alarm probability ($P_{f}$). $P_{d}$ and $P_{f}$ can be expressed as follows:

(2) $$\begin{array}{l} P_{d}=P\{H_{1}|H_{1}\}\\ P_{f}=P\{H_{1}|H_{0}\} \end{array}$$

## 3. STFT-RADN Spectrum Sensing Algorithm

**Figure 1:**
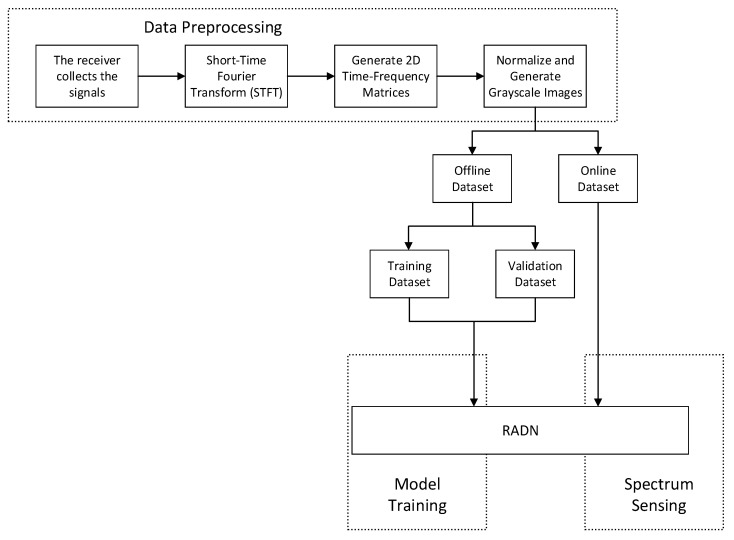
Spectrum sensing framework.

This paper adopts a deep-learning-based approach to address the spectrum sensing problem, utilizing RADN combined with STFT. The proposed spectrum sensing method consists of three main parts: data processing, model training, and spectrum sensing, as illustrated in Figure 1.

### 3.1. Short-Time Fourier Transform

To fully utilize the features of two-dimensional CNN, one-dimensional time series signals must be converted into two-dimensional matrix forms. Most existing deep-learning-based spectrum sensing methods generate two-dimensional grayscale images by segmenting and concatenating one-dimensional received signals. Using grayscale images as input aligns with the characteristic that most neural networks are more suitable for handling high-dimensional data, but this approach lacks the frequency-domain information of the signals.

By employing the STFT in signal processing, we can convert simple time-domain signals into feature spectra containing both time-domain and frequency-domain information [18]. This method generates two-dimensional grayscale images from received one-dimensional signals through time–frequency analysis, effectively reflecting the frequency-domain characteristics and providing strong robustness and noise resistance.

To efficiently extract signal features, this study employs STFT to convert time-domain signals into time–frequency representations. This representation captures the time–frequency characteristics of the signals, facilitating the distinction between signals and noise.

STFT breaks the signal samples into chunks using a window function, and each chunk is then discrete Fourier transformed (DFT) [19]. The mathematical expression for STFT is as follows:

(3) $$STFT(y(n))[t,\omega ]=\sum_{n=-\infty }^{\infty }y(n)w(n-t)e^{-j2\pi \omega n/N}$$

where y(n) is the input discrete-time signal, and N denotes the number of points in the DFT. The indices t and ω correspond to the time and frequency indices, respectively, and $w\left(n-t\right)$ represents the window function. Common window functions include rectangular, Hanning, Hamming, Gaussian, and Kaiser. Among these, the Kaiser window stands out due to its superior frequency characteristics, lower spectral leakage, and adjustable attenuation factor.

**Figure 2:**
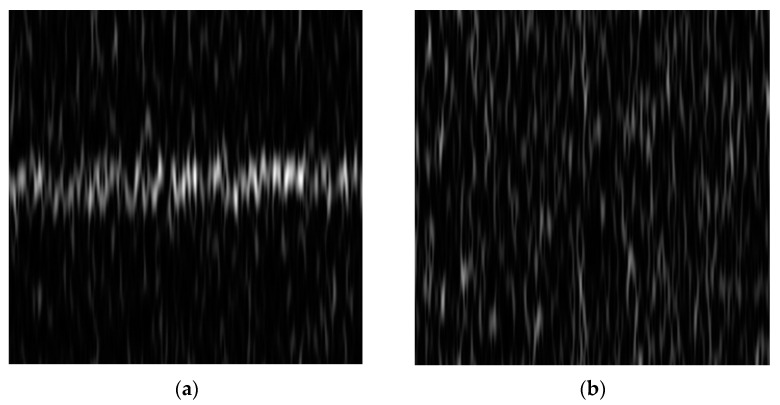
(**a**) Time–frequency image of $H_{1}$; (**b**) time–frequency image of $H_{0}$.

Through STFT conversion, the resulting time–frequency matrix can be represented as Y(t,ω). The matrix is then normalized to produce grayscale time–frequency images, as illustrated in Figure 2. The resulting grayscale images can be represented as follows:

(4) $$S(t,\omega )=\frac{Y(t,\omega )-\min(Y)}{\max(Y)-\min(Y)}$$

### 3.2. Convolutional Block Attention Module

The CBAM, proposed by Woo S et al. [20], is a representative model of hybrid attention mechanisms. It combines channel and spatial attention modules to adaptively optimize features by applying attention mechanisms to the feature maps of CNN, thereby enhancing the representation of important features. This effectively improves the network’s performance and generalization capabilities.

**Figure 3:**
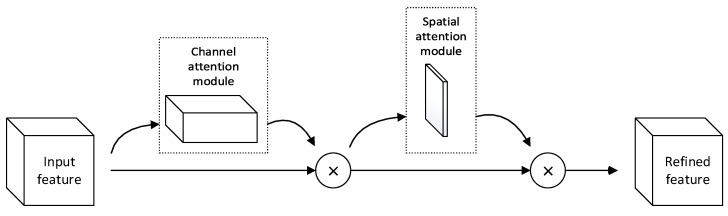
Convolutional block attention module.

CBAM consists of two submodules: the channel attention module and spatial attention module. These modules operate sequentially, enhancing feature representation by focusing on different feature channels and spatial locations, respectively. The overall structure of CBAM is shown in Figure 3, and its implementation process is described by Equations (5) and (6).

(5) $$F^{\prime }=M_{c}(F)⊗F$$

(6) $$F^{\prime \prime }=M_{s}(F^{\prime })⊗F^{\prime }$$

In the formulae, F represents the input feature map, $M_{c}$ denotes the channel attention weights, $M_{s}$ denotes the spatial attention weights, ⊗ represents element-wise multiplication, $F^{\prime }$ signifies the feature map after applying the channel attention weights, and $F^{\prime \prime }$ is the final output of CBAM.

**Figure 4:**
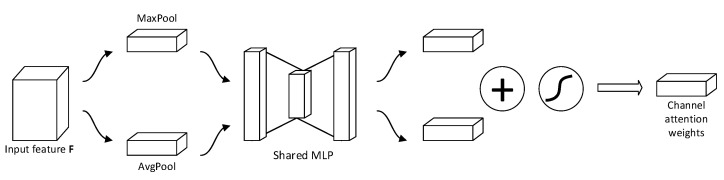
Channel attention module.

The goal of the channel attention module is to enhance important features by weighting different channels. The structure of the channel attention module is shown in Figure 4. First, the input feature map F undergoes global max pooling and global average pooling to aggregate spatial information. Then, the results of both global max pooling and global average pooling are separately fed into a shared multilayer perceptron (Shared MLP) to obtain two feature maps. Finally, the outputs of the MLP are combined using element-wise addition, and the resulting feature vector is passed through a sigmoid activation function to produce the channel attention weight matrix $M_{c}$.

The formula for the channel attention mechanism can be formulated as follows:

(7) $$\begin{array}{ll} M_{c}(F) & =\sigma (MLP(AvgPool(F))+MLF(MaxPool(F)))\\ & =\sigma (W_{1}{(W}_{0}{(F}_{avg}^{c}{))+W}_{1}{(W}_{0}{(F}_{\max}^{C})))\; \end{array}$$

where $F_{avg}^{c}$ and $F_{\max}^{c}$ represent the feature maps output by the average pooling and max pooling operations, respectively, and σ denotes the sigmoid function. $W_{0}$ and $W_{1}$ are the weights of the shared multilayer perceptron. $M_{c}(F)$ represents the output of the channel attention mechanism.

**Figure 5:**
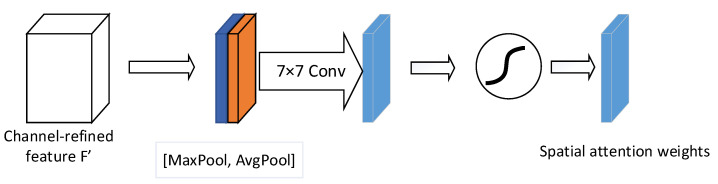
Spatial attention module.

The objective of the spatial attention module is to emphasize important spatial features by focusing on different locations within the feature map. The structure of the spatial attention module is illustrated in Figure 5. First, the input feature map $F^{\prime }$ undergoes global max pooling and global average pooling along the channel dimension, resulting in two separate feature maps. These feature maps are then concatenated along the channel axis. Finally, a convolution operation is applied to the concatenated result, followed by a sigmoid activation function to obtain the spatial attention weight matrix $M_{s}$.

The formula for the spatial attention mechanism can be formulated as follows:

(8) $$\begin{array}{ll} M_{s}(F^{\prime }) & =\sigma (f^{7\times 7}([AvgPool(F^{\prime });MaxPool(F^{\prime })])\\ & =\sigma (f^{7\times 7}\left[F_{avg}^{s};F_{\max}^{s}\right]) \end{array}$$

where $f^{7\times 7}$ represents the convolution operation with a kernel size of 7×7, and $M_{s}(F\prime )$ represents the output of the spatial attention module.

### 3.3. Res-Inception Attention Block (RAB)

**Figure 6:**
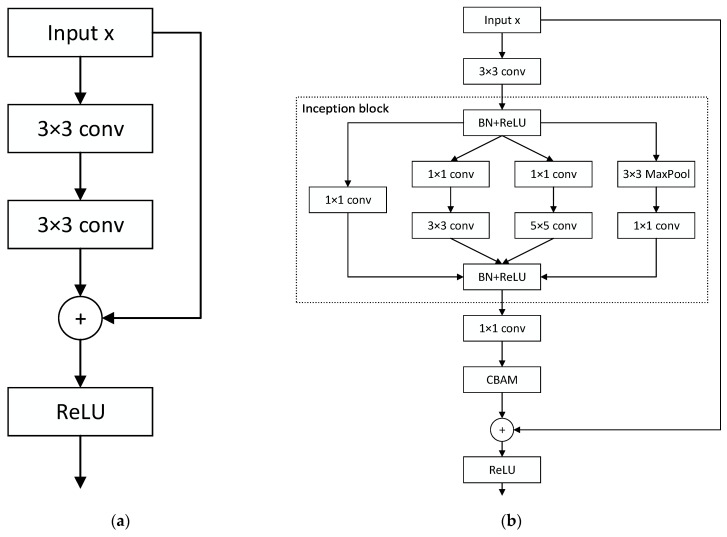
(**a**) Residual block; (**b**) res-inception attention block.

The residual block concept was first introduced by He et al. [21] to address gradient vanishing and exploding problems arising from increasing network depth. The core idea is to introduce shortcut connections for direct input propagation across layers, thereby improving deep network training and mitigating gradient vanishing or exploding issues. Additionally, residual blocks can effectively reduce overfitting in deep networks. The basic structure of a residual block is shown in Figure 6a.

However, the basic residual block has limitations in feature extraction capabilities. The inception module can simultaneously extract features at different scales, capturing richer image information [22]. The CBAM module can focus on important features in both channel and spatial dimensions, enhancing feature extraction. Therefore, this paper proposes the RAB, which incorporates the inception module and the CBAM module into the residual block, forming a more powerful feature extraction unit.

Furthermore, to improve feature extraction efficiency, this paper introduces batch normalization (BN), which accelerates the network’s convergence process, making the training more robust. The RAB is illustrated in Figure 6b.

The mathematical expression of the res-inception block can be formulated as follows:

(9) $$F\left(x\right)=f(x)+x$$

where x represents the input, f(x) denotes the residual mapping of the feature map, and F(x) is the output of the RAB. This design allows the RAB to extract multiscale features more effectively and capture key features within the image more efficiently. Additionally, by leveraging the advantages of the residual structure, this design significantly enhances the model’s performance while maintaining reasonable complexity.

### 3.4. Dense Group (DG)

**Figure 7:**
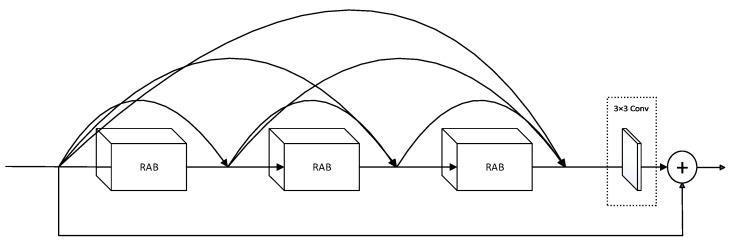
The structure of the dense group.

To further enhance the feature extraction capabilities of the RAB, this paper proposes a new fundamental module inspired by Zhang et al. [23]. This module forms a DG by stacking RABs and using a dense connection approach, as shown in Figure 7.

Stacking RABs allows the network to progressively extract richer and more abstract feature representations. This enables each block to learn new features and allows the network to capture more complex patterns and information.

Moreover, the dense connections link the output of each block to subsequent blocks, facilitating more efficient feature propagation and combination. This ensures maximal information flow between layers, promoting feature reuse. Additionally, adding a convolutional layer at the end of the DG enhances feature extraction, alignment, deeper feature learning, and eliminates information bottlenecks. This operation enhances the model’s performance, making it more efficient and accurate in handling complex tasks.

The mathematical expression for the DG is as follows:

(10) $$Y=G([X,F_{1}(X),F_{2}(X),F_{3}(X)])$$

where X represents the input feature map, $F_{i}(X)$ denotes the output feature map of the i-th residual attention block, and [⋅] represents the concatenation of multiple inputs. G(⋅) indicates the mapping of the feature maps, and Y denotes the output of the DG block.

### 3.5. Residual Attention Dense Network

**Figure 8:**
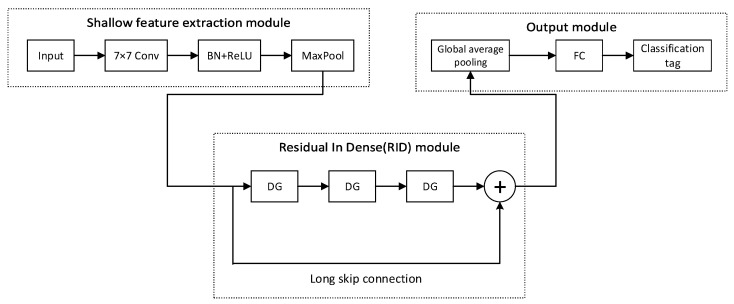
The structure of the RADN.

The proposed RADN consists of three parts: a shallow feature extraction module, an RID module, and an output module. First, the shallow feature extraction module captures basic features and reduces computational burden. Next, the RID module, consisting of densely connected residual blocks, enhances feature reuse and extraction capabilities, performs deep feature extraction, and improves gradient flow. Finally, in the output module, global average pooling aggregates information from the entire feature map, preventing overfitting. The features are then fed into a fully connected layer and classified using a Softmax classifier. The structure of RADN is shown in Figure 8.

In the STFT-RADN spectrum sensing algorithm, we selected n pairs of data $\left\{\left(S_{1},T_{1}),\ldots ,(S_{n},T_{n}\right)\right\}$ as the training set and m pairs of data $\left\{\left(S_{n+1},T_{n+1}),\ldots ,(S_{n+m},T_{n+m}\right)\right\}$ as the test set. Here, $S_{\cdot }$ represents the grayscale images obtained by applying the STFT and normalization to the received signals, and $T_{\cdot }$ represents the ground-truth classification label of the received signal. The input–output mapping of the whole network is as follows:

(11) $$f_{w,b}(S_{n})={\hat{T}}_{n}$$

where w and b are the trained weights and biases in the network, respectively, and ${\hat{T}}_{\cdot }$ represents the output mapping of $S_{n}$ after passing through the network. Due to the superiority of the cross-entropy loss function in binary image classification tasks, we have chosen it as our loss function. The cross-entropy loss function can be expressed as follows:

(12) $$Loss=-\sum_{i=1}^{2}T_{n}(i)\log({\hat{T}}_{n}(i))$$

Algorithm 1 provides a detailed explanation of the spectrum sensing algorithm based on STFT-RADN.
**Algorithm 1:** Spectrum Sensing Algorithm of STFT-RADN**Input:**Training and Testing Datasets**Output:**Detection probability $P_{d}$ and false-alarm probability $P_{f}$
**Data Collection:**Collect the signal samples X and preprocess the dataset into spectrograms S using STFT**Initialization:**Gaussian initialized weights θ and maximum iteration number IterMax
**Training:**Input the training set samples $\left\{\left(S_{n+1},T_{n+1}),\ldots ,(S_{n+m},T_{n+m}\right)\right\}$ While i≤IterMax=200: a. Update θ by performing backpropagation with an Adam optimizer on the loss function b. Increment i by 1 Save the model with lowest loss Until maximum epochs (IterMax = 500) reached**Online Testing:**Apply the trained RDAN model to the test dataset $\left\{\left(S_{n+1},T_{n+1}),\ldots ,(S_{n+m},T_{n+m}\right)\right\}$ and output the classification results Calculate the $P_{d}$ and the $P_{f}$


## 4. Experimental Results and Discussion

### 4.1. Experimental Environment and Data

To validate the performance of the proposed STFT-RADN spectrum sensing algorithm, this section presents the relevant simulation experiments and analysis of the results. The simulations were conducted on a system equipped with an Intel(R) Core(TM) i7-13650HX CPU and a GeForce RTX 4060 GPU.

We used MATLAB 2023a to generate five types of primary user signals: BPSK, QPSK, 8PSK, 64QAM, and 16QAM. The SNR range was set from −20 dB to 10 dB, with intervals of 2 dB. For each modulation type and SNR level, 1000 samples were generated, with a sampling rate of 200 kHz.

Before performing STFT time–frequency analysis to obtain the spectrograms, additive white Gaussian noise (AWGN) was added to the signals, and spectrograms without primary user signals were generated based on a Gaussian distribution. The experiment also considered Rician multipath fading to simulate the random attenuation of wireless signals in a multipath propagation environment.

The obtained STFT time–frequency dataset was divided into training, validation, and testing sets in a ratio of 7:2:1.

### 4.2. Effect of Sampling Points

**Figure 9:**
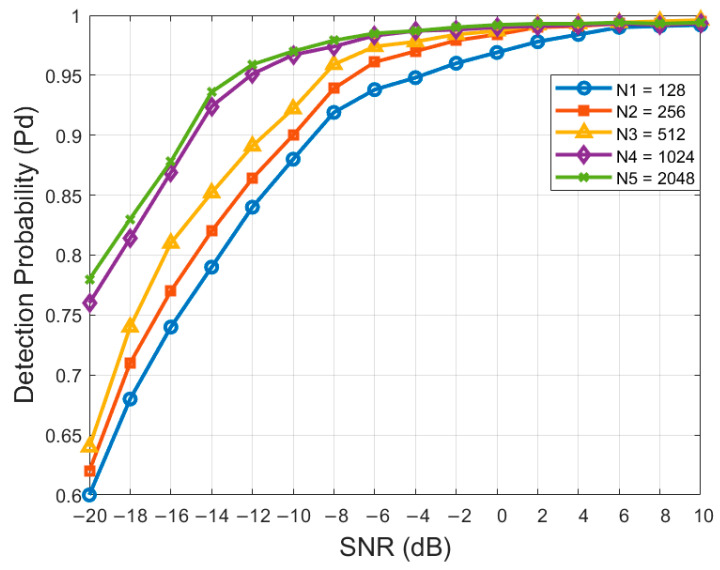
Effect of sampling points.

In this experiment, the detection probability of the STFT-RADN spectrum sensing model with different sampling points N is compared with the change in SNR. The experimental results are shown in Figure 9.

The experimental results show that detection probability increases with the number of sampling points. More sampling points result in a more significant improvement in detection probability under low SNR conditions (e.g., −20 dB to −10 dB). This indicates that more sampling points provide richer information, aiding in better signal detection under low SNR conditions.

However, more sampling points also mean more input data, leading to increased computational complexity and longer detection times. When the number of sampling points reaches 1024, the growth trend slows down. This is because too many sampling points can cause inconsistencies in the information before and after sampling, making it difficult for the network to learn more effective and relevant information.

Balancing computational complexity and detection probability, we chose 1024 as the optimal number of sampling points.

### 4.3. Effect of Window Function

**Figure 10:**
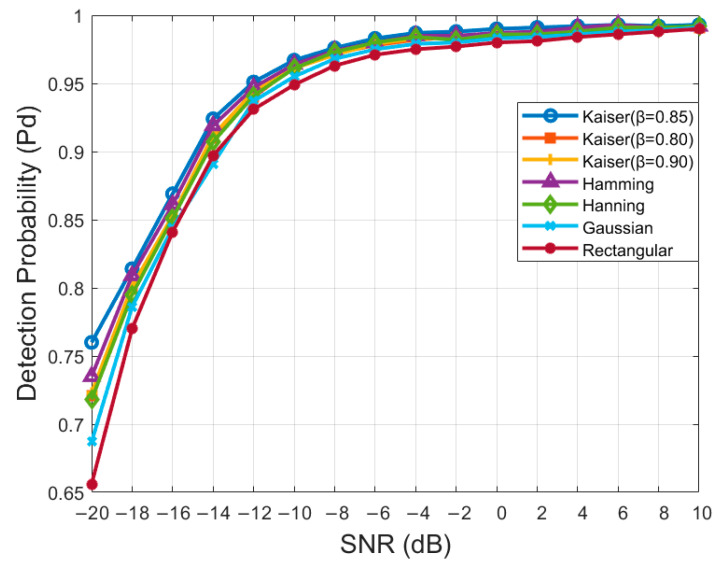
Effect of window function.

In this experiment, we compared the detection probability of the STFT-RADN model using various window functions. We conducted experiments at various SNR levels using several window functions, including the rectangular, Hanning, Hamming, Gaussian, and Kaiser windows with different attenuation factors (β). The experimental results are presented in Figure 10. The results indicate that the Kaiser window with β=0.85 provides the best overall performance, particularly under low SNR conditions, followed by the Hamming window. The Gaussian and rectangular windows demonstrate relatively poor performance. Consequently, this study adopts the Kaiser window with β=0.85.

### 4.4. Effectiveness of STFT

**Figure 11:**
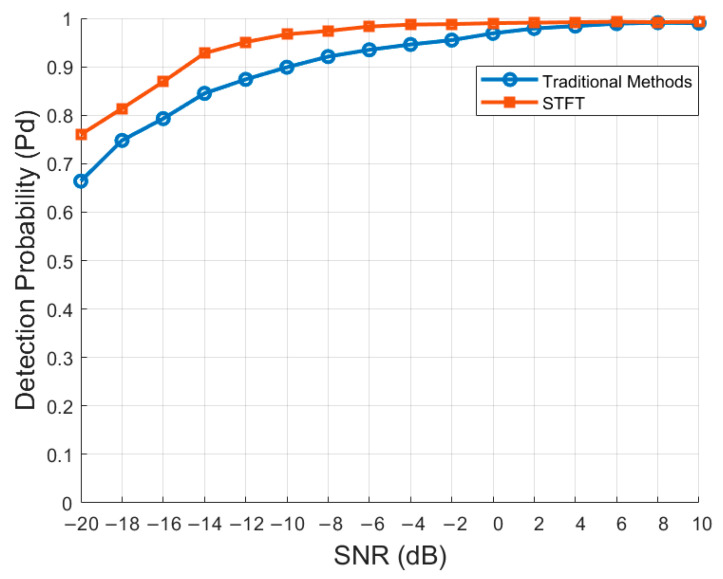
Effectiveness of STFT.

In this experiment, we compared the detection probability of datasets processed using traditional methods (cutting and splicing) [24] with that of datasets processed using STFT in the RADN model. The experimental results are shown in Figure 11.

The experimental results show that the STFT processing method significantly improves detection probability at low SNR values compared to traditional methods, with increases of 8.6%, 13.5%, 9.3%, 5.2%, and 3.5% at −20 dB, −14 dB, −10 dB, −4 dB, and 0 dB, respectively. This improvement occurs because STFT converts time-domain signals into the time–frequency domain. Even at low SNRs, the time–frequency representation provided by STFT can reveal the underlying signal structure, improving detection accuracy. This conversion helps capture both the temporal and spectral characteristics of the signal, enhancing detection and classification performance.

This demonstrates that using STFT to process datasets in the RADN model effectively captures signal information at low SNRs, thereby improving the overall performance of spectrum sensing.

### 4.5. Comparison of Detection Probability

**Figure 12:**
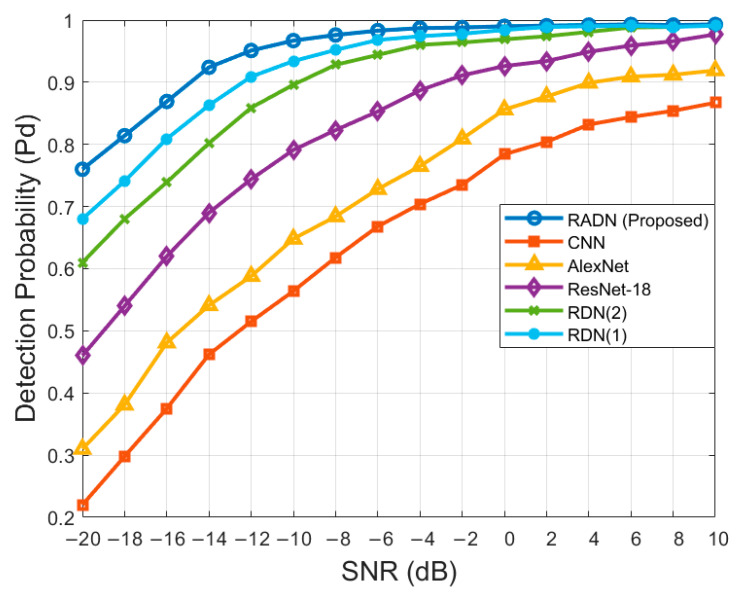
Comparison of detection probability.

In this experiment, we used the same dataset to compare the proposed RADN model with other state-of-the-art models. We evaluated the performance of our method against CNN [25], AlexNet [26], ResNet-18 [21], RDN (1), and RDN (2). Here, RDN (1) refers to the RADN model without the CBAM module, and RDN (2) replaces the res-inception block in RDN (1) with a basic residual block. The $P_{d}$ at different SNR levels was evaluated with $P_{f}=0.01$. The experimental results are shown in Figure 12.

The figure shows that the RADN, RDN (1), and RDN (2) models perform significantly better than traditional CNN, AlexNet, and ResNet models, demonstrating the superior feature extraction capabilities of the RID structure.

Compared to ResNet, the introduction of dense connections in RDN (2) improves the overall detection probability by approximately 8% in the range from −20 dB to 0 dB. Additionally, replacing the residual block in RDN (1) with the res-inception block results in a further 6% increase in detection probability between −20 dB and −10 dB. This improvement indicates that the res-inception block effectively enhances feature extraction capabilities under low SNR conditions.

The best-performing model overall is our proposed RADN network, which incorporates the CBAM attention mechanism into RDN (1). Compared to RDN (1), RADN improves the detection probability $P_{d}$ by approximately 5% between −20 dB and −10 dB. This enhancement is attributed to the CBAM module’s attention mechanism, which helps to focus on important features and thus improves performance under low SNR conditions. Therefore, the RADN model demonstrates a significant advantage in spectrum sensing tasks, maintaining a high detection probability under low SNR conditions and markedly improving overall spectrum sensing performance.

### 4.6. Comparison of ROC

**Figure 13:**
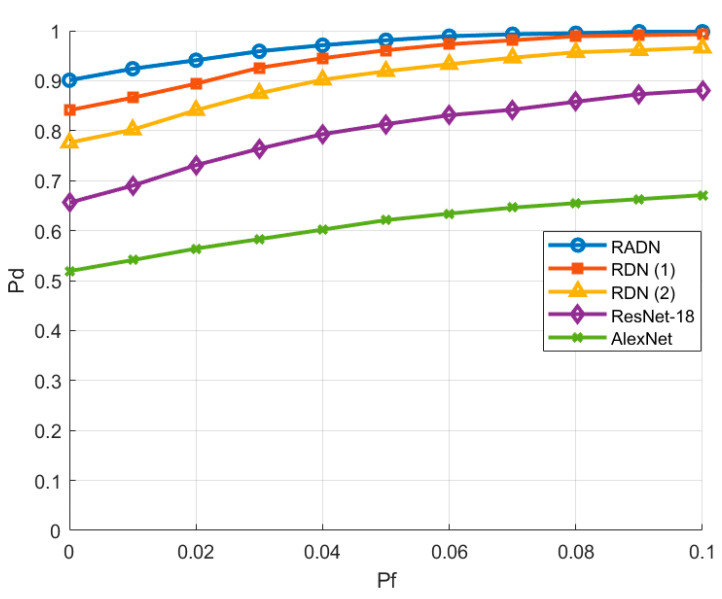
ROC curves of different algorithms.

To further evaluate the detection performance of RADN, we recorded the $P_{d}$ and $P_{f}$ from a series of extensive spectrum sensing experiments. By comparing the proposed RADN with RDN (1), RDN (2), ResNet-18, and AlexNet, we generated receiver operating characteristic (ROC) curves. All experimental results were conducted at an SNR of −14 dB, and the results are shown in Figure 13.

The experimental results indicate that with a constant SNR, $P_{d}$ increases with $P_{f}$ for all models. However, our proposed RADN network consistently exhibits a higher $P_{d}$ at the same $P_{f}$ compared to other models. For instance, when $P_{f}$ is 0.01, the $P_{d}$ of RADN is 5.8%, 12.2%, 23.4%, and 38.3% higher than that of RDN (1), RDN (2), ResNet-18, and AlexNet, respectively. Therefore, the experiments demonstrate that our proposed RADN model exhibits superior performance in spectrum sensing tasks.

### 4.7. Impact of Different Modulation Schemes on Detection Performance

**Figure 14:**
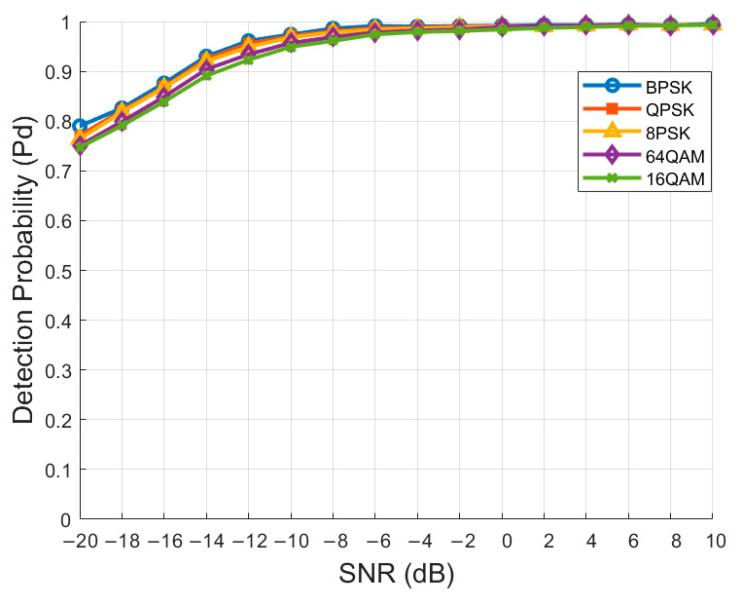
Performance of different modulation schemes in RADN.

In this experiment, we evaluated the performance of different modulation schemes under RADN, including BPSK, QPSK, 8PSK, 64QAM, and 16QAM. The experimental results are shown in Figure 14. The figure shows that RADN maintains a consistent $P_{d}$ across various SNR levels for different modulation schemes (BPSK, QPSK, 8PSK, 64QAM, 16QAM). This consistency indicates that RADN can handle different modulation schemes without significant performance degradation. It demonstrates that our proposed method is insensitive to the modulation order, making it suitable for various modulated signals.

### 4.8. Comparison of Efficiency

To validate the sensing efficiency of the STFT-RADN algorithm, we compared it with CNN-based, STFT-ResNet-18, and RADN algorithms using the same dataset. Table 1 presents the offline training time and online sensing time for each algorithm.

Experimental results indicate that while the STFT-RADN algorithm has slightly higher training and sensing times than traditional CNN and RADN methods, it reduces these times compared to STFT-ResNet-18. Based on the experimental results mentioned above, the STFT-RADN method slightly increases detection time compared to RADN without STFT preprocessing, but significantly improves $P_{d}$ under low SNR conditions. Although the STFT-RADN method incurs higher training and sensing times than traditional CNN, it significantly enhances detection probability, with the additional detection time remaining within an acceptable range. Furthermore, RADN outperforms STFT-ResNet-18 in both effectiveness and detection probability. Therefore, the STFT-RADN method demonstrates strong sensing efficiency, making it suitable for real-time detection.

### 4.9. Discussion

The experimental results clearly demonstrate that the proposed STFT-RADN spectrum sensing method outperforms traditional CNN-based methods. It effectively overcomes limitations such as inadequate feature extraction and poor performance in low SNR conditions. However, the increased complexity of RADN and the use of STFT come with trade-offs, particularly in terms of higher computational demands. This method requires greater processing power and memory than traditional approaches. In practical applications, especially in resource-constrained environments, striking a balance between computational efficiency and detection performance is crucial for real-time spectrum sensing. Future research could focus on optimizing the RADN architecture or developing lightweight neural networks that maintain high performance while reducing computational requirements.

## 5. Conclusions

This paper proposes a spectrum sensing method based on STFT and RADN. Unlike existing deep-learning-based spectrum sensing methods that directly extract features from one-dimensional signals, the STFT-RADN spectrum sensing method utilizes STFT to transform received signals into time–frequency spectrograms. This approach better captures signal characteristics, thereby enhancing detection performance. The proposed RADN model introduces the CBAM attention mechanism and improves the basic residual block. By combining the advantages of dense connections and residual connections, it forms a powerful deep feature extraction structure known as RID. This significantly enhances the network’s feature extraction capabilities and improves spectrum sensing performance. Experimental results demonstrate that the STFT-RADN spectrum sensing method significantly improves performance under low SNR conditions compared to traditional deep-learning-based methods. This method achieves a high detection probability and strong robustness and adapts to various modulation schemes, indicating broad application prospects. Future work will explore the application of RADN in multiuser cooperative spectrum sensing methods.

## Figures and Tables

**Table 1:** Comparison of efficiency.

Algorithm	Training Time/s	Sensing Time/s
CNN	26.44	3.14
STFT-ResNet-18	38.41	5.34
STFT-RADN	32.58	3.74
RADN	31.84	3.65

## Data Availability

Data are contained within the article.
